# Employer Adoption of Evidence-Based Chronic Disease Prevention Practices: A Pilot Study

**Published:** 2008-06-15

**Authors:** Jeffrey R Harris, Jeffrey Cross, Peggy A Hannon, Eustacia Mahoney, Sarah Ross-Viles, Alan Kuniyuki

**Affiliations:** Health Promotion Research Center, Department of Health Services, University of Washington; National Home Office, American Cancer Society, Atlanta, Georgia; Health Promotion Research Center, Department of Health Services, University of Washington, Seattle, Washington; Great West Division, American Cancer Society, Seattle, Washington, and National Home Office, American Cancer Society, Atlanta, Georgia; Health Promotion Research Center, Department of Health Services, University of Washington, Seattle, Washington; Health Promotion Research Center, Department of Health Services, University of Washington, Seattle, Washington

## Abstract

**Background:**

We conducted a pilot test of American Cancer Society Workplace Solutions, an intervention that takes a marketing approach to increasing employers' adoption of evidence-based practices to prevent and control chronic diseases among their employees.

**Context:**

We delivered the intervention and assessed the changes in practices of 8 large employers in the Pacific Northwest.

**Methods:**

Workplace Solutions recommends 15 employer practices in 5 categories: 1) health insurance benefits, 2) policies, 3) workplace programs, 4) health-promoting communication, and 5) tracking of employee health behaviors to measure progress. The intervention includes 4 meetings with employers over 2 months and begins with a questionnaire-based assessment of employer practices. Tailored recommendations follow, along with practice-specific implementation assistance on requested topics. We tested the intervention in a before–after study without a comparison group.

**Consequences:**

The employers ranged in size from 7500 to 115,522 employees and included private companies and public employers. Seven of the eight employers implemented more of the recommended practices at follow-up (an average of 13 months after the intervention) than at baseline. Overall, implementation of the practices increased from 38% at baseline to 61% at follow-up (*P* = .02).

**Interpretation:**

Workplace Solutions is a promising new approach to bringing evidence-based best practices for preventing chronic disease to large numbers of adults.

## Background

Employers are important community partners for preventing chronic diseases for 3 reasons. First, they have power over workplace environments that affect the lives of working-age adults, most of whom are in the workplace most days of the week (1). Second, employers face rapidly mounting health care and productivity costs attributable to chronic diseases experienced by their employees — many of whom are from the baby-boom generation and are now advancing through middle age (2) — and have increasingly strong motivation to promote preventive practices aimed at these diseases. Third, employers control health-insurance coverage of preventive care for 59% of working adults and their dependents (3). Recent research shows that employers do a poor job of covering evidence-based preventive care. For example, less than 10% of employers of any size offer optimal coverage for smoking cessation treatment (4).

For information about preventing chronic diseases among their employees, employers can draw on systematic reviews and recommendations from 4 expert groups: 1) the U.S. Preventive Services Task Force (USPSTF), 2) the Advisory Committee on Immunization Practices (ACIP), 3) the Task Force on Community Preventive Services (TFCPS), and 4) the Partnership for Prevention (PFP). The USPSTF and the ACIP review the effectiveness of clinical preventive care (5,6). The TFCPS and its Guide to Community Preventive Services review the effectiveness of policies, systems approaches, and community-based (including workplace-based) programs (7). The PFP builds upon the work of the USPSTF and the ACIP by prioritizing effective clinical preventive care services on the basis of cost-effectiveness and their impact on health (8).

Identifying and recommending best practices in health insurance benefits and workplace policies and programs for employers is the first step; increasing the adoption of these practices by employers is the essential next step. To ensure adoption, Maibach et al suggest using a marketing approach with 3 components: 1) conducting consumer research, 2) building sustainable distribution channels, and 3) improving products and product selection and reducing product price (9). Our research among workplace-related "consumers" has found that large employers and their human resources staff are important targets both because of the substantial number of people they employ and their potential to change norms regarding insurance coverage for preventive care (10). Our sustainable distribution channel is a large, voluntary public health organization, the American Cancer Society (ACS), which has long-standing relationships with employers, thousands of staff throughout all 50 states, and one of the best-recognized "brands" for health in the country. We followed Maibach's recommendation for improving products and product selection and for reducing product price in 3 ways. First, we selected a set of employer best practices that are evidence-based and focused on creating a supportive workplace environment for prevention. Second, we tailored the product to the needs and current practices of each employer. Third, we presented our information in face-to-face sessions in the workplace, emphasized the business case for cost-effective prevention, and provided implementation assistance to ease adoption for employers.

We present here the results of a pilot test of Workplace Solutions, a marketing approach to increase employers' adoption of evidence-based practices to prevent and control chronic diseases among their employees. Our purpose in conducting the pilot test was to assess the feasibility of our approach and to test whether employers adopted the recommended practices.

## Context

We conducted the study as a joint project of the ACS Great West Division, 1 of 13 geographic divisions of the ACS, and the University of Washington Health Promotion Research Center (HPRC), 1 of 33 Prevention Research Centers supported by the Centers for Disease Control and Prevention (CDC). ACS staff approached employers with whom they had established fundraising relationships about participating in this new workplace health promotion project. Employers were eligible if they had more than 5000 employees and headquarters in Idaho, Oregon, or Washington.

## Methods

### Selection of best practices

To select which best practices to include in our intervention, we reviewed the recommendations of the USPSTF, ACIP, and TFCPS for interventions applicable to the workplace, working-age adults, and prevention of cancer and other chronic diseases. We included best practices applicable to the workplace even if they had not been evaluated in the workplace. Our review produced a set of 15 best practices that we categorized into 5 functional groups: 1) insurance benefits, 2) workplace policies, 3) workplace programs, 4) tracking, and 5) communication (Table 1). Of the 15 best practices, 10 relate to creating a supportive environment for prevention.

### Intervention design

We tested the intervention in a before–after study without a comparison group. Our intervention consisted of 4 face-to-face meetings with each employer during 2 months. Our intervention team consisted of ACS staff accompanied by 1 or 2 members of the HPRC (J.R.H. and J.C.). We met with the employers' human resources staff in charge of purchasing health insurance benefits at each employer's headquarters. At the first recruitment meeting, we presented our general approach and emphasized the importance of preventing cancer and chronic diseases for employers and employees. At the second meeting, we measured the employers' baseline practices. After the second meeting, we wrote a 5- to 8-page report of recommendations for improving all practices that the baseline survey indicated were not fully implemented. At the third meeting, we presented the recommendations, discussed the potential for adoption of best practices, and asked the employers to choose 3 to 5 practices for adoption from the recommendations. At the fourth meeting, we presented Solution Sets for the practices employers selected. Solution Sets consisted of 1 to 3 pages of implementation-oriented text (i.e., a summary of evidence detailing why the practice should be adopted and information about how to implement the practice) and other supporting materials, cost calculators that estimated first-year implementation costs and return on investment, lists of vendors that could assist with the recommended practice, and information on relevant programs or materials available from the ACS (11).

Our intervention materials emphasized the business case for prevention of chronic diseases. For example, we highlighted that the PFP review rated 3 of the 5 clinical preventive services we recommended as either cost-saving (e.g., tobacco cessation treatment) or cost-neutral (e.g., colon cancer screening, influenza vaccination) (Table 1) (8) and that other analyses show that providing tobacco-cessation treatment and influenza vaccination to employees is usually cost-saving, particularly when productivity gains are counted (12,13).

The institutional review board of the University of Washington reviewed the study and classified it as exempt.

### Study measurements

Employers completed 3 questionnaires during the intervention period: 2 at baseline and 1 after the intervention. At baseline, each employer completed a pre-assessment survey of employer characteristics, employee demographics, and employers' insurance providers. Employers also completed a baseline survey of best practices, a comprehensive survey of the employers' health-related practices adapted from Golaszewski et al (14). The questionnaire included 115 items; 36 measured the 15 best practices that we included in our analyses. For follow-up at 1 year, we developed a streamlined version of the baseline survey and included only the questions relevant to the 15 best practices and a few items measuring employer satisfaction with the intervention.

We scored employers on their responses to the questions related to each best practice. For questions concerning benefit coverage and tobacco-use restrictions, we used 3 possible scores: 1) a score of 0 if the practice was not in place at all, 2) a score of .75 if the practice was partially in place (i.e., covered with co-pay for benefits or smoking forbidden indoors), and 3) a score of 1 if the practice was fully in place (i.e., coverage with no co-pay for benefits or a campus-wide ban on tobacco use). We used a score of .75 (rather than .50) for practices partially in place to reflect the fact that, by covering most of the costs associated with cancer screening and smoking cessation medications or by forbidding smoking indoors, employers are significantly aiding employees’ health. For all other questions, we scored dichotomously, using a score of 1 for a practice that was in place and a score of 0 for a practice that was not in place. For each best practice, we created a summary score by summing the items measuring the practice and dividing by the number of items. Thus, we scored each best practice as being implemented from 0% to 100%. For each employer, we calculated an overall best practice score by summing the scores on the individual best practices and taking the mean.

### Data analysis

Because the number of participating employers was small, we conducted primarily descriptive analyses. We calculated mean scores and 95% confidence intervals at baseline and follow-up, and mean change in score for 1) each best practice and 2) the total of all 15 best practices. Because of the small sample size and non-normal distribution of the data, we assessed the significance of the median change in scores with nonparametric sign tests. We present mean scores for ease of interpretation, but all presented *P* values (α = .05) are from these sign tests of median change. We also examined change in baseline and follow-up scores separately for practices for which employers did or did not receive Solution Sets.

## Consequences

### Participating employers

Of the 10 employers we approached, 9 agreed to participate in the intervention and completed the baseline surveys. One company later became ineligible to participate because it was purchased by another company, so the final sample was 8 employers. We followed employers for an average of 13 months (range, 8–18 months) from baseline to follow-up assessment. Employers ranged in size from 7500 to 115,522 employees (median, 12,695; mean, 33,104; SD, 37,408), and represented various industries (Table 2).

### Baseline implementation of best practices

The employers' total baseline best practice scores ranged from 23% to 58% (Table 2), with a mean of 38% (Table 3). Employers varied considerably among practices at baseline; they were most likely to cover cancer screenings (78%) and impose smoking restrictions or bans (72%) (Table 3). No employers had sun-protection policies, required insurance providers to track delivery of preventive services, or gave reminders for preventive services.

### Follow-up implementation of best practices

Seven of eight employers improved their total best practice scores from baseline to follow-up (Table 2). Scores at follow-up ranged from 37% to 85% (Table 2), with a mean follow-up score of 61% (Table 3), a significant increase from baseline (*P* = .02). Employers achieved significant improvement from baseline to follow-up in the areas of covering tobacco cessation treatment (31% mean change, *P* = .03) and covering cancer screening (18% mean change, *P* = .03) (Table 3). The lowest mean change occurred for providing sun protection (0%) and providing physical activity facilities (8%).

Our duration of follow-up varied from 8 to 18 months, and benefit–design cycles for employers are often 12 months long; however, we found little difference in the change in practices for the 6 employers with at least 12 months of follow-up (25% change) and the 2 employers with fewer than 12 months of follow-up (20% change).

### Impact of Solution Sets

Employers' baseline scores were lower for practices for which they received Solution Sets (31%) than for practices for which they did not receive Solution Sets (41%), yet the follow-up scores for both groups of practices were essentially the same (63% for those given Solution Sets, 60% for those not given Solution Sets). Thus, employers' scores improved 32% for practices with Solution Sets (*P* = .02) compared with 19% for practices with no Solution Sets (*P* = .45) (data not shown). Employers were most likely to request Solution Sets for covering tobacco cessation treatment (n = 7) and for covering cancer screening (n = 7).

### Feedback from employers

Employers were generally positive in their ratings of the intervention. Seven of the eight employers would recommend the intervention to other companies, and 5 employers intended to participate in additional programs offered by ACS.

## Interpretation

Workplace Solutions was associated with a large and significant increase in implementation of evidence-based best practices aimed at prevention of cancer and other chronic diseases by large employers. At baseline, only 38% of our recommended practices were in place, so there was substantial room for improvement. At follow-up, 61% of recommended practices were in place. Seven of eight employers improved their implementation of best practices after the intervention.

Of the 15 prevention practices we addressed, 2 (covering tobacco cessation treatment and covering cancer screening) improved significantly. From these results, we speculate that employers find it easiest to change practices that can be outsourced, such as health insurance coverage, but we need to test larger numbers of employers to be certain. The large employers in our study were all self-insured, and changing health insurance coverage may be more difficult for midsized and small employers that are not self-insured.

We found a large change among best practices for which we provided implementation-oriented assistance via Solution Sets. Furthermore, we found that employers were more likely to request this assistance for practices on which they scored poorly at baseline. These results suggest that more intensive intervention may have been associated with greater effect. Alternatively, the request for a Solution Set could have represented an intention by the employer to adopt the practice.

Our more subjective assessment of contributors and barriers to success raised 3 important points. First, age-appropriateness of our focus on chronic diseases could have been an issue for the one employer who made no change in response to the intervention. This employer had a much younger employee population than the other employers in our study. Second, the opportunity to work with high-ranking human-resources staff appeared to affect adoption of recommended practices positively. Third, our 2 government employers had relatively low rates of adoption of recommended practices, which may be caused by the long decision chain within unionized governmental bureaucracies.

The change in tobacco-related practices merits special mention. Like other researchers, we found that employer coverage of tobacco cessation treatment was low at baseline (4). Tobacco cessation treatment remains a missed opportunity for employers, because it is cost-saving and valuable to smoking employees. Unfortunately, it is not provided by most employers.

The limitations of this pilot study include its small sample, its design, and our focus on employer practices rather than employee behaviors. Our small pilot study included only 8 employers. Nonetheless, our intervention was associated with a meaningful and statistically significant change in the implementation of recommended practices.

Our study design, before–after without comparison, raises the possibility of historical effects, socially desirable responses, and interviewer bias. Changes of this magnitude during this short period seem likely to be due to the intervention rather than to historical effects. The objective nature of the practices we measured makes report of better practices because of social desirability unlikely. Interviewer bias is possible, because the intervention team measured implementation of practices both before and after the intervention. However, during interviewer training, we emphasized a consistent approach to measurement.

Our focus on employer practices and not employee behaviors was another limitation. Our best practices were recommended by the USPSTF or the TFCPS because they are effective in increasing healthy behaviors. We can reasonably expect that employees will improve the behaviors targeted by the intervention practices, but this remains to be proven.

We have developed and pilot-tested a marketing-oriented approach to improving large employers' practices for preventing chronic diseases among their employees. The behaviors we targeted are tied to the leading causes of death in the United States (15). The practices we targeted have a strong evidence base and rank highly on effectiveness and cost-effectiveness (8). Strengths of our marketing-oriented approach include its focus on employers' need to control health-related costs and its emphasis on environmental approaches to behavior change.

How generalizable is a resource-intensive intervention like ours to midsized and small-sized employers? In Washington, a state of average population size, there are approximately 206,000 employers (10). Approximately 200 large employers (i.e., those with more than 1000 employees) employ 18% of the workforce, and 3500 midsized employers (i.e., with 100 to 999 employees) employ 30% of the workforce. Approximately 202,000 small employers (i.e., with 99 or fewer employees) employ the remaining 52% of the workforce. Working with a partner like the ACS, reaching large and midsized companies with this type of an intervention or a streamlined version we are now testing in partnership with the ACS, might be feasible. However, for small employers we need to consider other broad-reach approaches, such as an interactive tool on the Web, or work through powerful intermediaries, such as health insurance brokers.

In the future, we plan to confirm the results of this pilot study with a larger employer sample and a more robust study design. Future studies should test the effects of the intervention on employee behaviors, employee productivity, and employer health care costs.

## Figures and Tables

**Table 1:** Employers' Best Practices for Preventing Chronic Diseases, by Practice Type, 8 Pacific Northwest Employers, American Cancer Society Workplace Solutions Pilot Study, 2005–2006

Practice Type	Best Practice	Relevant *Community Guide* Recommendation(s)^a^	Relevant USPSTF Recommendation(s) and Prevention Priorities^a^[CPB/CE/Total Scores^b^]
Insurance Benefits	1. Provide full coverage for tobacco cessation treatments, including prescription medications, over-the-counter nicotine replacement therapy, and counseling.	Reduce out-of-pocket costs for tobacco-cessation programs	Tobacco-use screening and cessation intervention [5/5/10]
	2. Provide full coverage for breast, cervical, and colon cancer screenings.	Reduce out-of-pocket costs for breast cancer screening	Breast: mammography [4/2/6]Cervical: Pap smear [4/3/7]Colorectal: any of 4 tests [4/4/8]
	3. Provide full coverage for influenza vaccination.	Reduce out-of-pocket costs for vaccinations	Annual vaccination for adults aged 50 and older [4/4/8]
	4. Require health plans to send reminders to members and network providers about preventive health services.	Client and provider reminders for breast, cervical, and colon cancer screening and influenza vaccination	
	5. Require health plans to track delivery of preventive health services and send performance feedback to network providers.	Assess providers' delivery of recommended cancer screenings and influenza vaccination and give feedback	
Workplace Policies	6. Ban tobacco use at worksites.	Smoking bans and restrictions (to reduce environmental smoke)	
	7. Post ""Use the Stairs" reminder signs near elevators.	Point-of-decision prompts to increase physical activity	
	8. Provide facilities for physical activity.	Enhance access to physical activity facilities in combination with informational outreach	
	9. Make healthy food choices available and affordable.	Multicomponent interventions aimed at diet, physical activity, and cognitive change	
	10. Require and provide sun protection for employees who work outdoors.	Insufficient evidence for occupational settings, but recommended for adults in recreational settings	Currently under review by USPSTF
Workplace Programs	11. Sponsor a tobacco cessation quit-line, including nicotine replacement therapy.	Multicomponent interventions that include client telephone support to increase tobacco cessation	Tobacco-use screening and cessation intervention [5/5/10]
	12. Provide annual influenza vaccination on-site.	Enhance access to vaccinations, in combination with intervention to increase community demand	Annual vaccination for adults aged 50 and older [4/4/8]
	13. Offer a workplace physical activity program.	Individually adapted health behavior change to increase physical activity	
Tracking	14. Survey employees' health behaviors to track effectiveness of health promotion efforts.	NA	
Communication	15. Conduct targeted health promotion campaigns, focusing on key health behaviors and use of preventive health care.	Multicomponent interventions to increase vaccination; small media to increase screening for breast, cervical, and colorectal cancers; and one-one education to increase breast and cervical cancer screening	

USPSTF indicates United States Preventive Services Task Force; CPB, clinically preventable burden; CE, cost effectiveness; NA, not applicable.

a Summary of recommendations from the USPSTF (5) and the *Community Guide* (7), as well as health impact and cost-effectiveness scores from the Prevention Priorities (9).

b Possible scores for both CPB and CE range from 1 to 5, with 5 indicating greatest value. Scores in this column as cited in Maciosek et al (8). Empty cells in this column indicate practices that are not recommended by ACIP or USPSTF.

**Table 2:** Employer Characteristics and Chronic Disease Prevention Best Practice Implementation Scores^a^ at Baseline and Follow-Up, 8 Pacific Northwest Employers, American Cancer Society Workplace Solutions Pilot Study, 2005–2006

**Employer**	Industry	Number of Employees	Baseline Score, %	Follow-Up Score, %	Change From Baseline Score, %
1	Financial	51,000	43	85	42
2	Retail Trade	11,712	58	58	0
3	Government	13,000	42	59	17
4	Agriculture	7,500	33	56	23
5	Manufacturing	8,710	27	75	48
6	Government	115,522	37	52	15
7	Retail Trade	45,000	23	37	14
8	Manufacturing	12,390	39	71	32

a Calculated by adding the scores for all best practices and then dividing by the total number of best practices (14 was the denominator for employers without outdoor workers, because best practice 10 [promote sun protection] was not applicable to them; 15 was the denominator for employers with outdoor workers).

**Table 3:** Mean^a^ Scores for Implementation of Best Practices to Prevent Chronic Diseases at Baseline and Follow-Up, 8 Pacific Northwest Employers, American Cancer Society Workplace Solutions Pilot Study, 2005–2006

**Best Practice^b^**	Mean Score at Baseline, %(95% CI)	Mean Score at Follow-up, %(95% CI)	Mean Change in Score, % (Range)	*P* Value^c^
1. Cover tobacco cessation treatment	35 (14-56)	66 (35-97)	31 (0-75)	.03
2. Cover recommended cancer screenings	78 (71-86)	96 (88-100)	18 (0-25)	.03
3. Cover influenza vaccination	69 (44-93)	88 (76-99)	19 (0-100)	.25
4. Send preventive services reminders	0	38 (0-81)	38 (0-100)	.25
5. Track delivery of preventive services	0	50 (0-95)	50 (0-100)	.13
6. Have a tobacco ban	72 (46-98)	72 (46-98)	0	>.99
7. Have "Use the stairs" signs	13 (0-42)	25 (0-64)	12 (0-100)	>.99
8. Provide physical activity facilities	63 (28-97)	71 (39-100)	8 (0-33)	.50
9. Provide healthy food choices	31 (0-62)	50 (11-89)	19 (0-100)	.63
10. Promote sun protection	0	0	0	NA
11. Have a tobacco cessation quit-line	25 (0-64)	63 (19-100)	38 (0-100)	.25
12. Provide on-site influenza vaccination	63 (29-96)	81 (52-100)	18 (0-100)	.25
13. Have physical activity programs	25 (0-64)	63 (19-100)	38 (0-100)	.25
14. Track employee health behaviors	25 (0-64)	50 (5 -95)	25 (0-100)	.50
15. Use health promotion campaigns	30 (5-55)	50 (23-77)	20 (0-100)	.22
Total best practice score^d^	38 (29-47)	61 (49-74)	23 (0-48)	.02

NA indicates not applicable.

a Means rather than medians are presented for ease of interpretation of change in scores from baseline to follow-up.

b Best practices scored from 0 to 1.00.

c *P* values (α = .05) derived from 2-tailed nonparametric sign tests.

d Calculated by adding the scores for all best practices and then dividing by the total number of best practices (14 was the denominator for employers without outdoor workers, because best practice 10 [promote sun protection] was not applicable to them; 15 was the denominator for employers with outdoor workers).
