# Erratum, Vol. 5: Issue 3

**Published:** 2008-09-15

Because of an editing error, 2 *P* values in the article, "Employer Adoption of Evidence-Based Chronic Disease Prevention Practices: A Pilot Study," were expressed incorrectly. The *P* values are located in Table 3 of the manuscript and should have been expressed as "*P* > .99," not "*P* < .99." Corrections were made to our website on August 7, 2008, and appear online at www.cdc.gov/pcd/issues/2008/jul/07_0070.htm. We regret any confusion or inconvenience this error may have caused.

